# Yeast Gup1(2) Proteins Are Homologues of the Hedgehog Morphogens Acyltransferases HHAT(L): Facts and Implications

**DOI:** 10.3390/jdb4040033

**Published:** 2016-11-05

**Authors:** Cândida Lucas, Célia Ferreira, Giulia Cazzanelli, Ricardo Franco-Duarte, Joana Tulha

**Affiliations:** 1 CBMA—Centre of Molecular and Environmental Biology, University of Minho, Campus de Gualtar, 4710-054 Braga, Portugal; giulia.cazzanelli88@gmail.com (G.C.); ricardofrancoduarte@gmail.com (R.F.-D.); tulha.joana@gmail.com (J.T.); 2 School of Biology, Faculty of Biological Sciences, University of Leeds, Leeds LS2 9JT, UK; c.m.ferreira@leeds.ac.uk

**Keywords:** yeast, GUP, HHAT, morphogenesis, Hedgehog

## Abstract

In multiple tissues, the Hedgehog secreted morphogen activates in the receiving cells a pathway involved in cell fate, proliferation and differentiation in the receiving cells. This pathway is particularly important during embryogenesis. The protein HHAT (Hedgehog *O*-acyltransferase) modifies Hh morphogens prior to their secretion, while HHATL (Hh *O*-acyltransferase-like) negatively regulates the pathway. HHAT and HHATL are homologous to *Saccharomyces cerevisiae* Gup2 and Gup1, respectively. In yeast, Gup1 is associated with a high number and diversity of biological functions, namely polarity establishment, secretory/endocytic pathway functionality, vacuole morphology and wall and membrane composition, structure and maintenance. Phenotypes underlying death, morphogenesis and differentiation are also included. Paracrine signalling, like the one promoted by the Hh pathway, has not been shown to occur in microbial communities, despite the fact that large aggregates of cells like biofilms or colonies behave as proto-tissues. Instead, these have been suggested to sense the population density through the secretion of quorum-sensing chemicals. This review focuses on Gup1/HHATL and Gup2/HHAT proteins. We review the functions and physiology associated with these proteins in yeasts and higher eukaryotes. We suggest standardisation of the presently chaotic Gup-related nomenclature, which includes KIAA117, c3orf3, RASP, Skinny, Sightless and Central Missing, in order to avoid the disclosure of otherwise unnoticed information.

## 1. Introduction

The yeast *Saccharomyces cerevisiae* is a recognised model to enlighten higher eukaryotic molecular processes, including the ones underlying human pathologies, which are very far from microbial life [1]. Intuitively, we associate yeasts, as microbes, to a planktonic form of life. However, microbes in the wild live mostly as large communities of cells forming biofilms or colonies, the biology of which remains largely unknown [2]. A colony or a biofilm displays a *proto-tissue* complex behaviour [3]. In these communities, cells organise spatially, morphologically and functionally to ensure the survival of the group. This implies the outlying orchestrated differentiation and death of cells to complete an efficient colonisation of the substrate, be it the pulp of a fruit or the surface of medical devices.

Within the large multicellular communities of yeast, cells behave similarly to their higher eukaryotic counterparts. They are born, grow larger and age while replicating until they senesce and die. Alternatively, they may die young, following an apoptotic cell death programme [4,5], allowing the supply of nutrients to the inner layers of the group [4], located further from the nutrient richer environment. Additionally, yeast cells may differentiate, shifting from yeast into true or pseudo-hyphae. These differ morphologically and physiologically [6,7,8]. The differentiation shift promotes an invasive behaviour generally associated with strain virulence [9]. Therefore, in contrast to planktonic growth, the survival strategy is collective [2] and resembles tissues from higher eukaryotes. Accordingly, large populations of yeast cells are imbedded in an extracellular matrix (ECM) composed of polysaccharides [10] and a large proteome [11], including many representatives of higher eukaryote ECM key proteins [11].

In higher eukaryotes, long distance communication between cells is achieved by secreted signalling proteins, namely from the Hedgehog (Hh) or Wingless (Wnt) pathways. These ligands interact with specific membrane-resident receptors that trigger a downstream signalling pathway in the receiving cell. No yeast cell-to*-*cell molecular communication vehicle has been recognised so far, although ammonia gradients have been suggested to perform a role in the collective orchestration of cell behaviour [12]. Moreover, the secretion of quorum-sensing small chemicals has been described in yeasts and suggested to perform a role in the control of population density [13,14]. Concomitantly, neither Hh- or Wnt-like pathways have been described in yeasts. 

The maturation of the Hedgehog secreted morphogens involves intein self-splicing and C- and N-terminal lipidation (reviewed by [15]). In *S. cerevisiae*, there is only one recognised self-splicing protein that has a Hedgehog-like Hint domain, the endonuclease VDE (PI-SceI), which is required for gene conversion during meiosis [16]. VDE derives from the splicing of Vma1, the alpha subunit of the V-ATPase [17], whose human homologue has a role in human breast cancer cell invasiveness [18]. More importantly, the highly homologous yeast proteins Gup1 and Gup2, discovered by our group in 2000 [19], have two homologues in higher eukaryotes that are responsible for the Hh-secreted morphogen N-terminal palmitoylation (HHAT/Gup2) [20,21] and the negative regulation of the pathway (HHATL/Gup1) [22]. Gup1 and Gup2 are members of the MBOAT superfamily of membrane bound *O*-acyltransferases [23,24] and are present in all eukaryotic genomes sequenced to date. Their designation as GUP (Glycerol Uptake Protein) comes from their influence on the performance of glycerol active transport [19,25], which is actually accomplished by Stl1 [26], meaning that Gup proteins were often mistaken for the actual permease (e.g., [27]). In animal cells, the nomenclature is not consistent. In mouse, human or fly cells, both Gup1 and Gup2 are known by numerous aliases (Table 1). Such ambiguity generated the dispersion of experimental data on these proteins, as well as the under-interpretation of their roles. In yeasts, the Gup proteins have been implicated in a vast number of phenotypes from very diverse but fundamental biological/molecular processes, which will be revised in this work. In mammals and fly, the experimental data are scarcer and more recent. Here, we review the information available from *S. cerevisiae* and from another model yeast, the human commensal/opportunistic pathogen *Candida albicans*, and complement this with the role of Gup homologues in the regulation of Hedgehog ligand secretion in higher eukaryotes. Moreover, data on amino acid sequence comparison throughout model organisms are presented and a harmonising nomenclature is suggested.

## 2. Yeast Gup1 and Gup2 Proteins

### 2.1. Gup1 and Gup2 Are Members of the MBOAT Superfamily

*S. cerevisiae GUP1* was first associated with the recovery of glycerol from the medium for osmoregulation purposes [19]. In accordance, glycerol active transport is deficient in *GUP1*-deleted mutants [19,25]. However, this is an indirect effect, resulting from the involvement of Gup1 in lipid raft formation [37]. Raft disassembly causes Pma1 H^+^-ATPase plasma membrane misdistribution [27], decreasing the appropriate proton motive force for active transporters like Stl1 to work properly [37]. Gup1 has a close homologue Gup2. The two proteins share a high degree of similarity (77%) and identity (57%) [19]. Gup1 and Gup2 are members of the MBOAT superfamily of multispanning membrane-bound *O*-acyltransferases, a superfamily that was first suggested based exclusively on sequence similarity between a small group of proteins [23]. Besides Gup proteins from *S. cerevisiae*, the MBOAT superfamily includes proteins as diverse as mammalian acyl-CoA:cholesterol acyltransferases (ACAT), fly palmitoyltransferases (Porcn and Nessy), *Arabidopsis thaliana* diglyceride acyltransferases (DGAT), bacteria *Bacillus subtilis*, *Staphylococcus aureus* and *Pseudomonas aeruginosa* d-alanyltransferases (DltB) and alginate *O*-acetyltransferases (AlgI) [23]. All of these proteins share a highly conserved histidine residue localised in a hydrophobic domain (His^447^ in yeast Gup1), as well as another residue of histidine, asparagine or aspartic acid, localised 30–50 amino acids upstream, within a hydrophilic region (His^411^ in the yeast Gup1). These conserved positions specify the active centre of these enzymes. The MBOAT superfamily is presently subdivided into three functionally different subgroups. One group includes enzymes involved in neutral lipid biosynthesis (ACATs and DGATs), another group includes proteins involved in phospholipid remodelling (lysophosphatidate acyltransferases—LPATs) and a third group includes the enzymes implicated in protein acylation (Porcupine (from the Wnt pathway), HHAT(L) (from the Hh pathway) and Ghrelin *O*-acyltransferase—GOAT (from the insulin regulatory pathway)) [38]. Gup1, although included in the third group, has also been included in the LPATs group due to the multiple biological and molecular roles assigned to it. In the mammalian HHAT(L), the highly conserved MBOAT signature amino acid residues are different. The yeast Gup1 His^411^/His^447^ and Gup2 His^461^/His^498^ positions are occupied by aspartic acid and histidine in HHAT and by aspartic acid and leucine in HHATL, respectively. Interestingly, the substitution of His^447^ by a leucine in *S. cerevisiae* Gup1 caused loss of phenotype [39], raising the question whether mammalian HHATL actually functions as an acyltransferase [22]. The roles attributed to Gup1 in all cellular models are for now exclusively biological/phenomenological, not biochemical. In contrast, the HHAT from high eukaryotes has a fully recognised enzymatic function in the N-palmitoylation of the Hh-secreted signals that can be reproduced in vivo and in vitro [21,40]. The yeast Gup2 shares with Gup1 the two MBOAT conserved histidines in the amino acid sequence positions 460 and 496.

According to the most common algorithms used to calculate the hydrophobicity of amino acid sequences, the Gup1 and Gup2 proteins both have 10 well-defined transmembrane domains (TMD). This number of TMD implicates that Gup1 terminals are both located on the same side of the membrane. However, the Gup1 C- and N-terminal domains are localised, respectively, in the periplasmic and cytosolic sides of the membrane [41], which is a rather uncommon topology. HHAT from mammals has a re-entrant loop stabilised by a lipid attachment, a palmitoylation of the MBOAT aspartic acid signature (Asp^339^) [42]. This re-entrant loop actually comprises both MBOAT signature amino acids (*M. musculus* Asp^339^ and His^374^; *H. sapiens* Asp^340^ and His^377^). *S. cerevisiae* Gup1 and Gup2 and *C. albicans* Gup1 present a partially hydrophobic region, located between TMD 7 and 8 (using the TMD prediction engine available at [43]). The correspondent sequence aligns perfectly with the sequence from the *M. musculus* HHAT re-entrant loop, showing several conserved several amino acid residues. In silico palmitoylation prediction of Gup amino acid sequence (using the prediction engine available at [44]) indicates a fully conserved cysteine residue in that region: *S. cerevisiae* Gup1 Cys^396^ and Gup2 Cys^445^; *C. albicans* Gup1 Cys^417^; *M. musculus* HHAT Cys^324^ and HHATL Cys^326^; *H. sapiens* HHAT Cys^325^ and HHATL Cys^326^; and *D. melanogaster* RASP Cys^326^. This degree of conservation, in addition to the possibility that a palmitoylation is involved, suggests that Gup proteins might require the MBOAT signature residues to be embedded in or at the surface of the membrane, whether this is obtained by Cys or Asp palmitoylation as in mammalian HHAT [42]. This possibility will have to be studied in the future.

### 2.2. Gup1 and Gup2 Subcellular Localisation

In *S. cerevisiae*, Gup1 protein co-localises with markers of the plasma membrane, the endoplasmic reticulum (ER) and the mitochondria [19]. Gup2 shares the first two locations [45]. The more prominent localisation of Gup1 appears to be the plasma membrane, as revealed by GFP tagging at either N- or -C-terminal [41]. The plasma membrane localisation of Gup1 is dependent on the secretory pathway proteins Sec6-4 [41] and End-3-mediated excision [26,41]. In opposition, in mammalian cells (mouse myocytes cell line), the Gup1 orthologue localises predominantly in the cytosol and only residually in the ER closer to the nucleus [34]. 

### 2.3. GUP1 and GUP2 Expression in Yeast

The yeast *GUP2* promoter displays consensus sequences for 65 transcription factors (TF), and *GUP1* for 19 TF (Table 2; [46]). These include major players in yeast transcription control, like the general stress regulators Msn2 and Msn4, the glucose-repression controller Mig1, Ste12 from pheromone response and mating and Gcn4 from nitrogen-associated regulation. Three TFs are predicted to regulate both promoters identically (Tec1, Mig3 and Rap1), and one—Ash1—is predicted to activate *GUP1* and inhibits *GUP2* transcription (Table 2).

The presence of numerous TF consensus sequences in yeast *GUP1*/2 promoters could reflect a complex transcription regulation, yet *GUP1* and *GUP2* are almost invariantly expressed in several different conditions. This is the case for cells actively growing on glucose (repression conditions—fermentation) and on glycerol or ethanol (de-repression conditions—respiration) [47], as well as in cells adapted to osmotic stress conditions created by high amounts of salt [47,48], or subjected to osmotic shock by high amounts of sorbitol [49]. These results suggest that the expression of these genes might be constitutive [47]. Nevertheless, this cannot be the case since *GUP1* transcription duplicates upon NaCl shock [50]. Additionally, at the level of the protein, Gup1 is stably internalised in an End3-dependent manner upon sudden shift from glycerol to glucose [26,41]. Glycerol has multiple and complex roles in yeast cells. It is a respired poor carbon source whose metabolism requires induction and relief from glucose-repression. It functions as an osmolyte (reviewed by [51]), a lipid synthesis precursor [52], a *pendulum* of the NAD(H) redox balancing cytosol/mitochondrial shuttle [53], and is necessary for maintaining the signalling competent state of the cells [54]. The fact that Gup1 is retrieved from the plasma membrane upon glycerol-to-glucose transition, and overexpressed upon salt shock when glycerol accumulation is required for cell survival [51], establishes a connection between Gup1 and glycerol-associated regulation, which nature keeps unknown. 

*GUP2* is generally less expressed than *GUP1* [47], and *gup2∆* null mutants do not present any marked phenotype in response to carbon sources (glucose/glycerol) or stress (salts, ethanol, weak acids and high temperature). In contrast, *gup1∆* mutants are moderately sensitive to all stresses [28] with the exception of high temperature, to which *gup1∆* is highly sensitive [28,55]. The expression of *GUP2* (not *GUP1*) has been shown to increase considerably upon ultraviolet irradiation [56], leading the authors to suggest Gup2 as part of the Early Genotoxic Response, i.e., the response to metal poisoning.

Up-regulation of Gup1 induces the up-regulation of several genes resident in the ER and Golgi, from the secretory pathway and lipid synthesis [39]. Kar2, a key protein from the Unfolded Protein Response (UPR) signalling pathway, stands out for its 24-fold increased expression [38]. Up-regulation of Gup1 further induces proliferation of membranous cisternal structures, with 1–7 per cell, physically separated from each other. This phenomenon is dependent on Ire1, another protein from the UPR pathway. ER, Golgi and itinerant proteins are present in these structures. Gup1 was also found, appearing to diffuse in and out of these structures [39]. Remarkably, the production of these membrane structures is dependent on the H^447^ MBOAT signature amino acid.

## 3. Yeast Phenotypes Associated with the Deletion of *GUP1*

### 3.1. Phenotypes Emerging from Genome-Wide Yeast Screenings

The extensive list of phenotypes associated with the deletion of *GUP1* comprises many that were obtained in genome-wide screening. These include cytoskeleton polarisation and bud site selection patterns [57,58], secretory and endocytic pathways [59], vacuole morphology [59] and anaerobic growth [60]. Other high throughput studies cannot be presently rationalised, namely Askree et al. [61], which mentions Gup1 as an important protein in the maintenance of proper telomere length. Importantly, many of these studies show the resistance of the *gup1∆* mutant to pharmaceutical drugs such as the anti-tumoral imatinib [62], the cytostatic cisplatin [63], the anti-inflammatory ibuprofen [64], the immunosuppressant FK506 [65] and the antimicrobial thymol [66]. Further resistance associated with *GUP1* deletion includes lithium [67] and arsenic [68]. This large list, by itself, suggests an equally large complexity of Gup1 functions. In contrast, *GUP2* has been referred to in only two HTP screenings [69,70]. The few evidences show that *GUP2* expression is increased in response to ethanol stress in wine fermentative conditions/strains.

### 3.2. Cell Wall Integrity and Biogenesis

**Figure 1:**
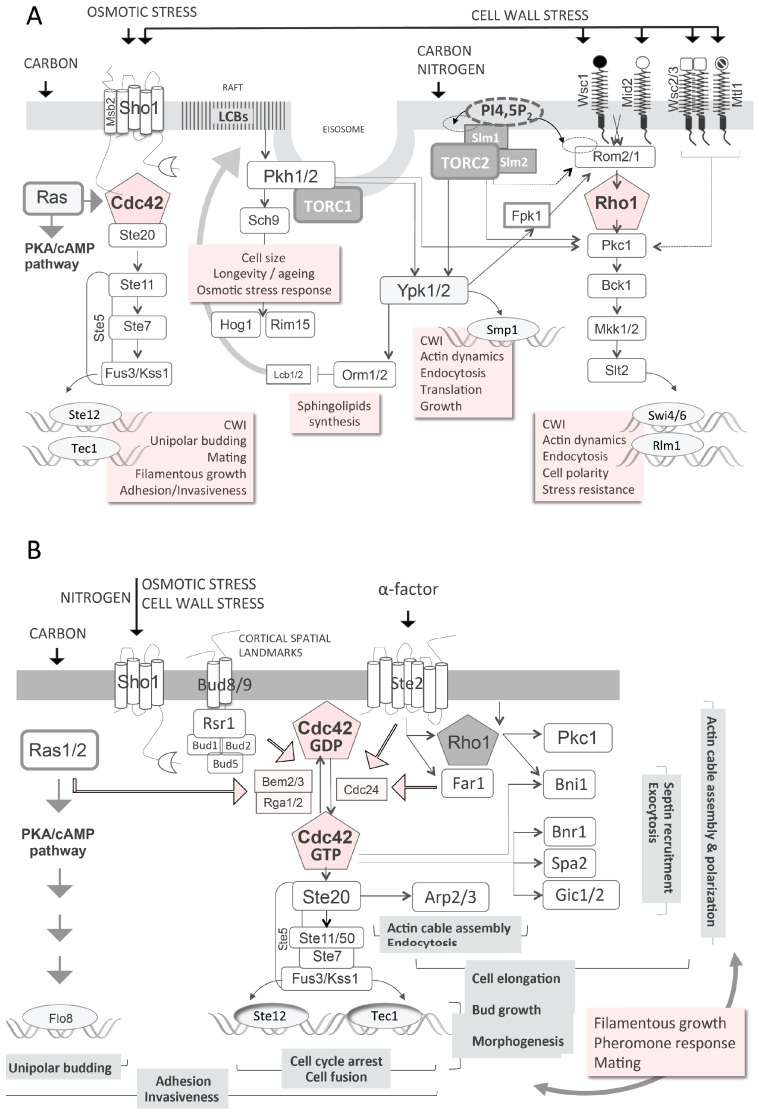
Major players implicated in the phenotypes associated with the deletion of *GUP1* in *S. cerevisiae* and *C. albicans* (grey boxes). Plain arrows indicate established interactions; dashed arrows indicate possible interactions. Pentagons refer to nodal proteins exerting multiple signalling, affecting positively or negatively many other proteins. (**A**) The Sho1 downward cascade belongs to the HOG (High Osmolarity Glycerol) pathway, and the Rom/Rho cascade constitutes the Cell Wall Integrity—CWI/PKC pathway. Crosstalk between HOG, PKC, the complex lipids and TORC1/2 signalling pathways relevant for *GUP1* associated phenotypes is shown; (**B**) Centrality of Cdc42 phosphorylation (pink boxes) in the control of pathways ultimately promoting differentiation, polarity, growth and reproduction. The central role of one protein shared by many pathways is designated in the text as *star*-type pathway.

As mentioned above, *gup1∆* is very sensitive to high temperature [28,55]. Generally, the sensitivity of yeast to high temperature is correlated with major defects in cell wall. Consistently, the deletion of *GUP1* in *S. cerevisiae* causes an increase in the cell wall content of β-1,3-glucans (+25%) [28] and chitin (+90%) [28,71], and a decrease in the amount of mannoproteins (−70%) [28]. These large differences are responsible for a deficient wall morphology and sensitivity to wall-perturbing agents [28]. In spite of these defects, the Cell Wall Integrity (CWI)/PKC pathway is signalling normally in the *gup1∆* mutant, as seen by MAPK (Slt2) (Figure 1A) dually phosphorylated state [28,72] after induction of the pathway by hypo-osmotic shock [28] or high temperature, or by NaCl or ethanol stress [73].

The CWI/PKC pathway controls not only the biogenesis of cell wall constituents and their remodelling, but also the associated organisation of the actin cytoskeleton and secretory pathway (reviewed by [74]). Unlike mammalian cells, *S. cerevisiae* only has one protein kinase C (Pkc1) that responds to the G-protein Rho1, the yeast homologue of RhoA. Rho1 activates Pkc1 besides other effectors. This allows the coordinated control of the actin cytoskeleton, exocytosis, membrane fluidity and the synthesis of wall glucans through transcription regulation in response to nutrients and stress [75]. Rho1 in turn is activated by Rom2/1 GDP/GTP exchange factors. These respond directly at least to a couple of wall stress sensors, Wsc1 and Mid2, and are further activated by the ligation of PI(4,5)P2 (phosphoinositol biphosphate) and/or of Slm1/2 proteins. The latter are targets for TORC2 (Target of Rapamycin 2 (TOR2) Complex) phosphorylation [76]. TORC2 also directly activates Pkc1, inducing the PKC pathway response. Importantly, Rho1 and consequently the PKC pathway are also activated by the α-factor plasma membrane receptor Ste2, a physical partner of Gup2 (Saccharomyces Genome Database). Rho1 is a signalling node, regulating many proteins and being under the effect of many others (including Kog1 from TORC1 (Target of Rapamycin 1 (TOR1) Complex) [77]), and it acts on actin cytoskeleton, thus causing cellular shape/polarity and filamentation [78].

TORC2 is redundant with TORC1 in the control of major cellular functions, but the cell wall composition and integrity and the polarisation of the actin cytoskeleton associated with cell growth, division and morphogenesis is only dependent on TORC2 [79,80]. Even though TOR proteins are very similar phosphatidylinositol kinase (PIK)-related kinases, only TOR1 is sensitive to inhibition by caffeine or the immunosuppressant rapamycin (reviewed by [80]). The *gup1∆* mutant, both alone and in combination with *gup2∆*, is resistant to rapamycin [73]. On the other hand, *gup1∆* is equally sensitive to caffeine as the wild type strain but, unlike this last, its sensitivity is remediable with sorbitol [28].

The mechanisms of action of caffeine and rapamycin are poorly understood. Both induce Rho1 activation and depend on this induction to inactivate the TORC1 pathway [77]. Caffeine induces the activation of Slt2 in a Tor1/Rom2 dependent manner [81] and elicits a transient decrease in the intracellular levels of cAMP, concomitantly inhibiting the Ras/cAMP pathway [81]. Moreover, caffeine supposedly activates the Pkc1 cascade through Tor1-mediated signalling [81]. Rapamycin, on the other hand, represses rRNA transcription and induces arrest of translation and cell cycle at G1. Furthermore, it causes glycogen accumulation, sporulation and autophagy [82]. These effects demand the complexation of rapamycin with Fpr1 (yeast equivalent of the immunophilin FKBP12) and subsequently with TOR1 [83]. Resistance to rapamycin in *S. cerevisiae*, can be provided by Fap1, which compets with Fpr1 [83,84]. However, the way in which Gup1 might be involved remains obscure.

Bearing in mind that the deletion of *GUP1* causes defects in cell wall [73], membrane [37,55] and endocytosis [59], the cause of resistance to rapamycin could be the inability of the drug to enter the cell. Rapamycin is practically insoluble in water and poorly soluble in ethanol or glycerol [85]. This, together with its large molecular mass (≈900 Da), suggests an endocytic entrance. Additionally, *gup1∆* is also resistant to the calcineurin inhibitor FK506 [65]. In mammalian cells, this immunosuppressant drug binds to FKBP12 inhibiting the Ca^2+^ signalling. Actually, FKBP12 bridges FK506, calcineurin and IP3 (inositol 3-phosphate) receptor, promoting the dephosphorylation of the latter, thereby modulating Ca^2+^ flux and signalling [86]. In yeast, FK506 affects the Ca^2+^-mediated response to high temperature, high NaCl-osmotic stress and α-factor pheromone [87]. High temperatures and high sodium, as discussed above, cause severe phenotypes in *gup1∆* [19,28], while α-factor secretion is higher in *gup1∆* [59].

The regulatory roles of phosphoinositides in yeast are not as well understood as in higher eukaryotes [88]. Still, their synthesis and turnover pathways are genetically characterised and the effects of some of the enzymes on major cellular processes have been recognised. At least seven unique second-messenger molecules are generated that regulate growth, differentiation, cytoskeletal rearrangements, membrane trafficking and protein vacuole sorting [88]. The phosphoinositide signalling operates in yeast as a *star-type* pathway, i.e., by cross-talking with numerous other pathways controlling their associated transcription, thus promoting the orchestrated cellular response to numerous stimuli. Major effectors associated with phosphoinosides in yeast belong to TORC2 and PKC pathways.

Yeast TOR pathways respond to carbon and nitrogen availability [89]. In silico analysis of the promoter regions of both *GUP1* and *GUP2* unveiled a series of putative regulatory sequences, from which the presence of repeated consensus sequences for Gcn4, a major transcription factor that responds to amino acid starvation and nitrogen in the dependence of TORC1, stands out [90]. Accordingly, wild-type strain and *gcn4∆* mutant expressing lacZ reporter constructions for *GUP1* and *GUP2* promoter regions showed that β-galactosidase activity depended on Gcn4 for full expression of the *GUP1* but not *GUP2* in synthetic medium. If the medium is supplemented with 0.5% ammonium sulphate and 15 mM glycerol, the expression of Gup1 in *gcn4∆* reverts to the values of wild-type strain [73]. The *gup1∆* mutant dual dependence on nitrogen source and glycerol suggests an intricate position of the Gup1 protein in the midst of crossroads of N and C regulatory pathways. Moreover, the operationality of PKC signalling observed in the *gup1∆* mutant suggests that the TORC2 pathway is not affected by the function of Gup1 protein. However, as discussed below, many of the cellular functions controlled by TORC2 are defective in the absence of *GUP1*. Besides wall damage-associated phenotypes, the most striking of these is the altered polarity during division exhibited by *gup1∆* mutant [57,58].

The *gup1∆* was found to display profoundly aberrant random bud site selection [57,58,76]. This implies a large defect on the proper establishment of cytoskeleton polarity [91,92], which is known to depend on Rho1/Pkc1 signalling [78]. It is as well dependent on other pathways associated with the multiple roles of the signalling node Cdc42 [93] (Figure 1B), including the Sho1 branch of the HOG pathway [94]. Cdc42 has the same fundamental role in mammalian cells (Figure 1B). This intricate crosstalk implicates the coordinated control of actin cytoskeleton remodelling for directional growth (elongation), which is mandatory for cell division through budding or mating. However, more importantly, underlies the morphogenic switch into filamentation that mediates invasion and adhesion [6,95].

### 3.3. High Osmolarity Glycerol (HOG) Pathway

**Figure 2:**
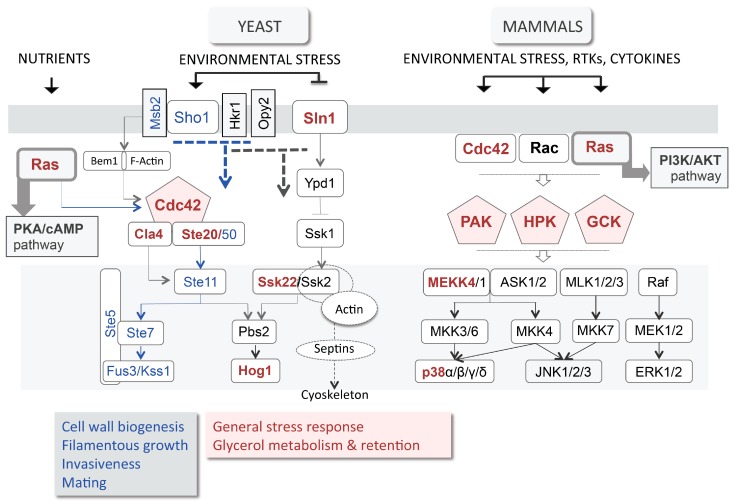
Parallelism between MAPK signalling cascades in yeast and in mammalian cells. In bold: proteins that functionally complement yeast mutants. In orange: proteins that are identical in yeast and mammalian pathways. In blue: proteins involved in the yeast phenotypes affected by *GUP1* deletion. Pentagons refer to nodal proteins exerting multiple signalling, affecting positive or negatively many other proteins (*star-type* pathway). Required nomenclature disambiguation: PAK: P21 Activated Kinase; HPK: Histidine Protein Kinase (Sln1 is also a histidine-aspartate phosphotransferase/kinase); GCK: Germinal Center Kinase; MEKK4 = MTK1.

In spite of the *GUP1* deletion, the CWI/PKC pathway is working properly at the level of Slt2 phosphorylation when stimulated by hypotonic shock [28]. Nevertheless, the wall of the mutant is severely affected. Two other pathways contribute to the wall integrity and remodelling: the Sho1 branch of the HOG pathway, and the Long-Chain Base (LCB)/sphingolipids YPK pathway (Figure 1A). HOG stands for High Osmolarity Glycerol, a name derived from control of the production and accumulation of the yeast osmolyte glycerol in response to high osmotic stress (reviewed by [51]). The HOG pathway controls many other processes such as polarity, adhesion and invasiveness, filamentation (i.e., differentiation), mating and cell wall biogenesis (reviewed by [96]). Importantly, the HOG pathway is functionally equivalent to the mammalian Wnt/p38 stress responsive MAPK pathway (Figure 2) [97,98]. Null mutants from HOG pathway have been successfully complemented by murine [99] or human p38 and associated kinases [100,101]. The p38 pathway is a non-canonical branch of Wnt MAPK, as are the JNK and ERK5 branches. Nevertheless, it has been shown to crosstalk with the Wnt canonical ERK1/2/β-catenin branch [102]. Actually, the four Wnt branches (ERK1/2, p38, JNK and ERK5) are all partially redundant in the control of growth/proliferation, i.e., cell cycle progression, differentiation, adhesion, migration and apoptosis in response to growth factors, cytokines and stress (reviewed by [103,104]). The HOG pathway Sho1 sensor functions in an exceptionally complex manner [105]. It binds to effectors that deliver different responses: Msb2 binds to Cdc42, activating the Ste11/Ste7/Fuss3/Kss1 filamentation/mating and cell wall biogenesis pathway, and Hkr1 or Opy2 that activate the HOG pathway (Figure 1 and Figure 2) [105,106].

The strains without *GUP1* are unable to grow under high osmotic stress [19,28]. This could indicate a malfunction of the HOG pathway and a concomitant deficient production and/or accumulation of glycerol. Hyperosmotic stress also stimulates the CWI/PKC pathway [107], a fact that is not surprising since the adaptation to high osmotic stress implicates changes in cell volume and turgor that demand remodelling of the cell wall. The simultaneous activation of HOG and CWI/PKC pathways can also result from other stimuli, such as high temperature [108] and the action of zymolyase, an enzyme cocktail with β1,3-glucanase activity [109,110]. *gup1∆* is extremely sensitive to both of these stimuli [28]. The effect of zymolyase is particularly severe, namely in comparison with lyticase, another wall disrupting enzyme [28]. This severity could be the consequence of the increased percentage of β1,3-glucans in the mutant cell wall. The malfunction of HOG rather than CWI/PKC pathway was also inferred from the pronounced phenotype of *gup1∆* mutant in the presence of Calcofluor White [28] compared to Congo Red, which caused a negligible defect [111]. While some insults to the cell wall like Congo Red only trigger CWI/PKC [81,112], others trigger both HOG and CWI/PKC pathways [107,108,109,110]. In spite of the intricate interplay between both pathways (reviewed by [107]) the phenotypes caused by *GUP1* deletion associated with osmoregulation [19] and the cell wall [28] are consistent with a relation of Gup1 with both pathways.

### 3.4. Plasma Membrane Composition and Associated Signalling

Gup1 is implicated in several membrane and lipids associated phenotypes [37,55,60,113]. The signalling pathway associated with complex lipids, the LCB/sphingolipid YPK pathway (partially depicted in Figure 1A), is not very well studied in yeasts, and no parallel has been established with the mammalian counterpart. The activation of Ypk1/2 is dependent on phosphorylation by Pkh1/2 kinases [114,115] or by TORC2 [115,116]. Pkh1/2 responds to LCBs and is implicated in membrane trafficking events. These include endocytosis through Pkc1-associated actin remodelling [114], protein phosphorylation [117], the trafficking of glycosylphosphatidylinositol (GPI)-anchored proteins, and the cell surface expression of amino acid permeases [118]. In mammalian cells, sphingosine acts as an inhibitor of PKC, or as activator of casein kinase II, Pkcζ, Pak1 (p21-activated kinase) and PDK1 (phosphoinositide-dependent kinase 1) (reviewed by [119]). 

The lipid membrane composition and rafts content have implications in membrane fluidity/rigidity. In this regard, membrane fluidity homeostasis has been suggested to be under the control of a novel pathway that involves Rho1 effector [75]. According to these authors, the overlap between the CWI pathway with the membrane fluidity homeostasis pathway includes at least six components: Rho1, Pkc1, Bck1, Mkk1, Mkk2, Slt2 and Swi6. This is comparable to the overlap between mating response, pseudohyphal growth and the HOG pathways [107]. Membrane fluidity depends ultimately on the amount of complex lipids. Accordingly, the depletion of sphingolipids elicits the activation of TORC2 through Ypk1/2 and Orm1/2 that in turn stimulate their synthesis (Figure 1A) [120]. 

Down-regulation of TORC2-Ypk1 signalling allows cell survival under high osmotic stress independently of HOG pathway activation [121]. TORC2-Ypk1 signalling operates through closing of the glycerol channel Fps1, by blocking its phosphorylation by Ypk1. This is crucial to maintain the Fps1 channel open in the absence of stress [121]. This TORC2-Ypk1-mediated control occurs independently of the control of the channel by Hog1 [122]. Moreover, the enzyme responsible for deviating the glycolytic flux towards glycerol production Gpd1 (cytosolic glycerol 3 phosphate dehydrogenase) is also a target for Ypk1/2 [122], while its mitochondrial isogene Gpd2, from the glycerol NAD(H) shuttle, is under the direct control of glucose and is activated by phosphorylation via the AMP-activated kinase Snf1 [122]. Moreover, glycerol consumption and active uptake through Stl1 permease [26] is severely affected by the absence of *PKC1* [123]. This strengthens the notion that glycerol is a major driver in maintaining the signalling competent state of cells, as suggested before [54], namely in the crossroads of responses to nutrients availability and stress through TORC2, sphingolipids, CWI/PKC and HOG pathways. The defects caused by *GUP1* deletion appear in the centre of this puzzle, suggesting a pivotal role in signalling for the Gup1 protein.

LCB/sphingolipids form Detergent-Resistant Microdomains (DRM) in the plasma membrane, also known as lipid rafts due to their particular molecular/physicochemical properties. These properties derive from the predominantly saturated hydrocarbon tails of the sphingolipids and the high concentration of sterols: cholesterol in animal cells (e.g., [124]) and ergosterol in yeasts [125,126]. In yeast, as in mammalian cells, lipid rafts participate in protein sorting (contributing to the functions of transporters, pumps, sensors, receptors or G-proteins), dynamically including or excluding proteins [127,128,129]. Importantly, the components of lipid rafts are also actively involved in signalling and in stabilization of actin cytoskeleton–membrane interactions and therefore polarity [128,129]. 

In yeast, many proteins are known to functionally localise in lipid rafts forming a complex patchwork [128], which biogenesis is still poorly known. The first proteins found in yeast membrane microdomains were the H^+^-ATPase pump Pma1 [130] and the amino acid permease Can1 [131]. Presently, almost 300 proteins have been found to differentially localise into lipid rafts [128], including many permeases involved in ion homeostasis, water, glycerol or drugs transport as well as sugar or nitrogen transport/sensing, cell wall metabolism, stress response, signalling, flocculation, bud site selection and eisosome formation. Some are GPI-anchored like the β1,3-glucanosyltransferase Gas1 [125], while others are prenylated, or farnesylated and palmitoylated like Ras2 [132], but most just have transmembrane domains (see [128] for a comprehensive list). Different types of membrane lipid microdomains were identified and designated identified on the basis of the proteins they harbour: Membrane Compartment occupied by Pma1 (MCP), Can1 (MCC) [131] or TORC2 (MCT) [133]. 

The plasma membrane localisation of Gup1 [19,41] and Gup2 [45] has not been associated with raft residence [128]. Still, a patchy distribution of the labelled Gup1 protein was observed by electron microscopy [41]. Importantly, *gup1∆* is affected in lipid rafts integrity and assembly [37]. This was evidenced by the even, not punctate, distribution of sterols in the mutant plasma membrane, as well as the 40% lower amounts of DRM recovered [37]. Additionally, Pma1 (H^+^ pump known to reside in lipid rafts, specifically MCPs [128]) is absent from *gup1∆* DRMs, and the amounts of Gas1 (the most abundant yeast GPI-anchored protein) are residual, suggesting that rafts were unselectively affected by *GUP1* deletion [37]. Accordingly, Gup1 interferes in sterol and sphingolipids synthesis, as evidenced by respectively increased and decreased resistance of the mutant to inhibitors of specific biosynthetic steps [37].

The yeast cell is a facultative anaerobic organism that can efficiently endure environments with virtually inexistent oxygen. In these conditions, the yeast cell does not synthesise sterols. Instead, it must take up sterols from the environment using the Pdr11 ATP-binding cassette (ABC) multidrug transporter [134], another permease known to locate in lipid rafts [128]. *GUP1* was found to be crucial for survival in anaerobiosis [63]. In *gup1Δ*, not only is the biosynthesis of sterols affected, but also the uptake of sterols is impaired, underlying its inability to grow in anaerobic conditions [60].

Yeast lipid rafts assembly, unlike mammalian counterparts, takes place in the ER, only subsequently stepping into the Golgi [125]. Moreover, MCPs and MCCs are likely to segregate differently, since Pma1 is only incorporated into the rafts outside the ER [130]. ER resident proteins targeted to sphingolipids/ergosterol-poor membranes, such as the vacuole membrane, are not involved in rafts assembling [129]. Therefore, the defect in rafts assembly generated by *GUP1* deletion must occur in the ER (one of Gup1 localisations) and may induce the segregation of specific membrane/protein assemblages towards their correct location. Another group of results concurs for a clear implication of the Gup1 protein in the secretory/endocytic pathways. In yeast, the vacuole performs the role of mammalian lysosomes. Proteins are addressed to the vacuole for degradation by the same pathway as carboxypeptidase Y (CPY), through the late endosome (reviewed by [135]). *GUP1* was identified as one of the genes required for efficient sorting of CPY to the vacuole, as well as for the maintenance of this organelle regular size and morphology [59]. Its deletion also causes the excessive secretion of α-factor [59], the ER-resident protein Pdi1 [28], and the GPI-anchored β1,3-glucanosyltransferase Gas1 [37].

**Figure 3:**
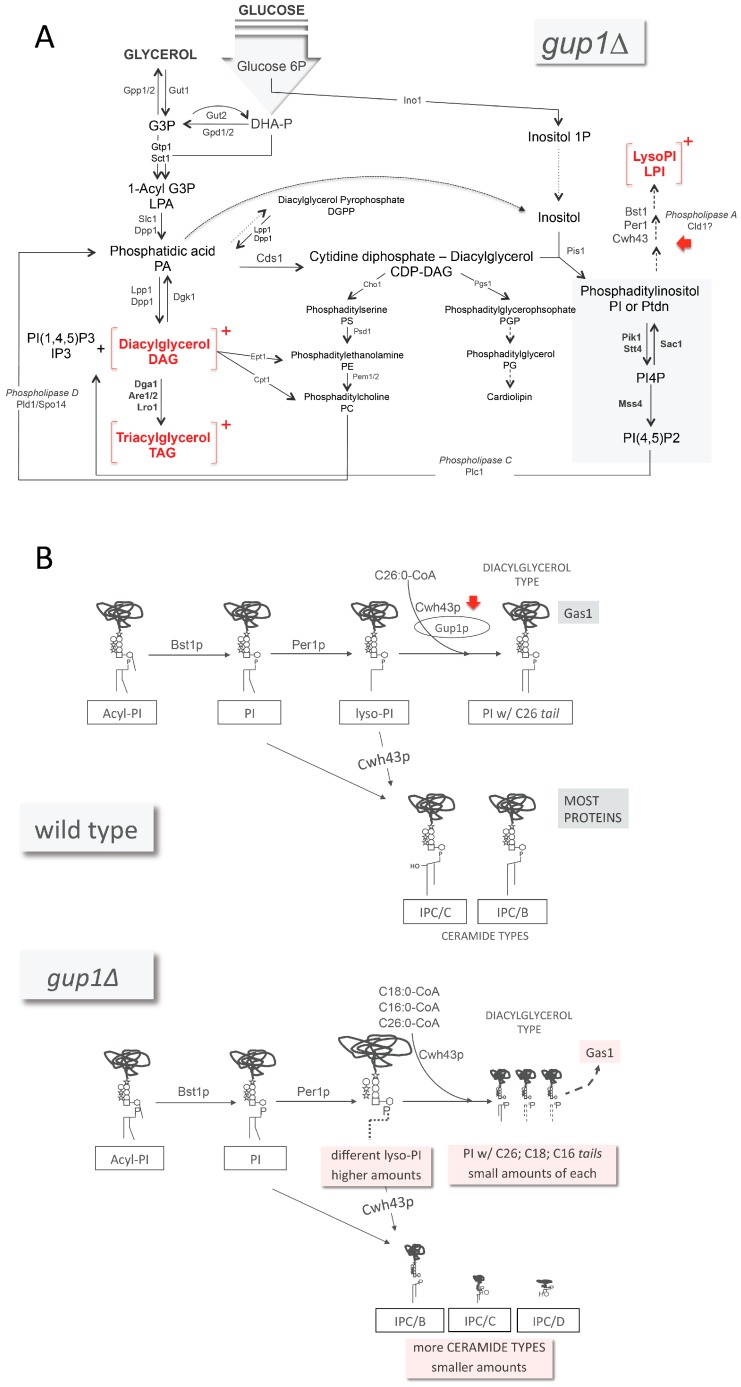
(**A**) Key lipid synthesis pathways from *S. cerevisiae*. In red are shown the types of lipids which amounts are affected by the *GUP1* deletion. Steps in GPI anchor synthesis occurring in *S. cerevisiae* wild-type strain (**B**) and in *gup1∆* mutant (C). Gas1 is an example of a GPI anchored protein that is found excessively liberated into the medium in the mutant cultures. Red arrows indicate the step in which Bosson et al. (2006) suggest that Gup1 acts as an acyltransferase. GPI anchor backbone is composed of mannose (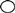), glucosamine (✩) and ethanolamine-P (☐). Dark thick scrawls represent peptide chains. PI: phosphatidylinositol; IPC: inositol phosphoceramide. Types B, C and D were defined from bands obtained using TLC [136].

**Figure 4:**
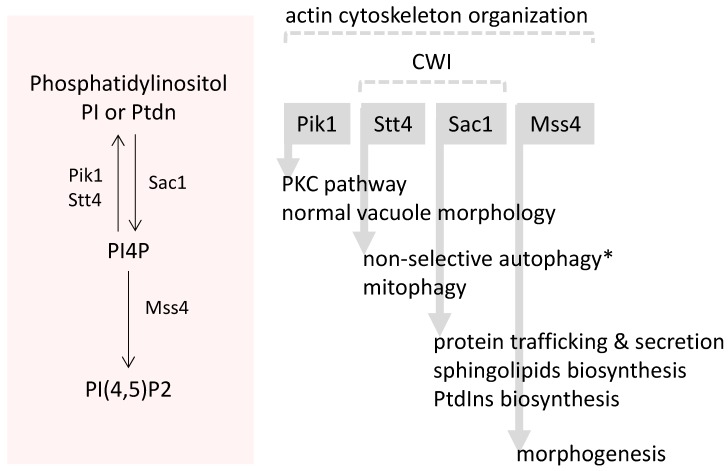
Phenotypes associated to the enzymes known to catalyse the steps from PI to PI(4,5)P2 in *S. cerevisiae*. These phenotypes are also associated with the *GUP1* deletion, with the exception of mitophagy. * Preliminary results [45].

The deletion of *GUP1*, besides affecting sterols and sphingolipids synthesis and impairing sterols uptake, also causes changes in the regular concentrations of major lipid types: a high increase in diacyl- and triacylglycerol (Figure 3A) and a decrease in phospholipids [55]. Fatty acids synthesis, on the other hand, remains normal [37]. However, Gup1 is not recognised as an enzyme from lipid biosynthesis pathways. In this regard, Bosson and co-workers [113] proposed the involvement of Gup1 in the GPI synthesis/remodelling process, which occurs at the level of the ER in *S. cerevisiae* [136,137]. The anchors of mature GPI-anchored proteins of *S. cerevisiae* contain either ceramide or diacylglycerol with a C26 fatty acid in the *sn*2 position [138,139]. Gup1 was proposed to be the enzyme that adds C26 fatty acids to the *sn*2 position of lyso-phosphatidylinositol (lyso-PI)-containing GPI anchors (Figure 3A,B) [113]. Accordingly, the *GUP1* deleted mutant, in contrast to the wild-type strain, was shown to accumulate lyso-PI and to contain phosphatidylinositol tails with C16 and C18 fatty acids instead of C26, all found in much smaller amounts compared to wild type (Figure 3B) [113]. The direct involvement of Gup1 as an enzyme in this process is nevertheless controversial. The wild-type GPI-anchor types can still be found in the mutant [113]. Concomitantly, it was suggested that Cwh43 was able to catalyse a bypass to the Gup1-dependent step in inositol phosphoceramide (IPC) *tail* maturation (Figure 3B) [140,141], using indifferently PI or Lyso-PI as substrates (Figure 3B). Moreover, the metabolic step associated with Gup1 is preceded by the transformation of PI into lyso-PI by Per1 (Figure 3B) [139]. In *gup1∆* mutants, the lyso-PI harbours different fatty acid chains, suggesting that the Per1-dependent step is affected by the deletion of *GUP1* as well [113]. Altogether, it is prudent to restrain from naming Gup1 as a mere GPI remodellase, since this classification does not take into account the multiple phenotypes associated with the protein, neither does it consider the localisation of Gup1 in the plasma membrane and its association with the mitochondria. In view of the complexity associated with Gup1 protein so far, the effects observed in GPI anchor synthesis could be indirect. GPI-anchor remodelling, if confirmed to be a direct function of Gup1 as an enzyme, will be among the other functions. The enzymes that catalyse the last steps of PI(4,5)P2 synthesis have associated phenotypes common to the *gup1∆* mutant (Figure 4) (Saccharomyces Genome Database). This could provide the rationale for considering the possibility of Gup1 acting upstream of these enzymes, at the level of PI production (Figure 3). A change in PI availability would provide a reason for the changes observed at the level of the ceramide and diacylglycerol types (Figure 3B). The inosite metabolism was though never studied in this mutant.

### 3.5. Cell Death

Apoptosis is the most common process of Programmed Cell Death (PCD) in eukaryotes. It is vital for the fast elimination of useless or injured cells and for the differential development of tissues and organs. The existence of PCD processes in yeasts is presently unarguable [142]. As mentioned above, multicellular aggregates of microbial cells, like colonies or biofilms, are spatially organised and require the specialization of cells differentially localised to ensure supply of nutrients and water to the whole cell ensemble [143]. PCD occurs in a differentiated way within these aggregates [4]. The underlying rationale is that older cells of the colony centre that have already multiplied extensively, dying can contribute to the survival of adjacent cells with limited access to nutrients and water [4].

Several types of stimuli, both endogenous and exogenous, are known to cause yeast cells to enter a PCD process. H_2_O_2_ and acetic acid are major exogenous triggers commonly used to induce apoptosis in yeast (reviewed by [142]). However, several additional agents have been reported to induce apoptotic phenotypes, namely, salt stress, ethanol, heavy metals, UV radiation and high temperatures. Endogenous triggers on the other hand, include defects in N-glycosylation, chromatid cohesion, mRNA stability and ubiquitination (reviewed by [142]). Moreover, DNA damage (resulting from oxygen metabolism and ROS generation) and replication failure can stimulate the activation of yeast cell death programmes. Apoptosis may also play a role in ageing, replicatively [144] or chronologically [145,146].

Gup1 is required for several cellular processes that are related to apoptosis, namely the above discussed rafts integrity and stability [37], lipid metabolism [55], including GPI anchor correct remodelling [113], mitochondrial [60] and vacuole [59,147] functions and actin dynamics [57,58]. Accordingly, the deletion of *GUP1* induces the hypersensitivity of yeast cells to two known apoptosis inducing conditions [148], chronological life span (CLS) [149] and acetic acid (reviewed by [150]). Two apparently contradictory works suggest that the mutation decreases [148], or extends [147] CLS. In this last work, replicative life span (RLS) is also described to increase in the absence of *GUP1*. The main difference between both studies resides in the amino acid availability in the yeast growth medium, which was four times higher in the study by Li et al. [147] than in the one by Tulha et al. [148]. Considering the above-mentioned suggestions that Gup1 might be implicated in ammonium and TOR signalling, which is controlled by nitrogen/amino acid abundance [79], the mutant will likely respond differently to apoptosis induction in either cultivation condition.

On the other hand, in the presence of lethal concentrations of acetic acid, *gup1Δ* unlike the wild type undergoes a necrotic-like cell death process [148]. This is inferred from the absence of typical apoptotic features: (a) maintenance of the membrane integrity, (b) phosphatidylserine externalisation, (c) depolarisation of mitochondrial membrane, and (d) chromatin condensation. Moreover, preliminary assays on autophagy inducing conditions, i.e., nitrogen starvation, show that the deletion of *GUP1* causes a clear decrease of autophagic activity as determined by GFP-Atg8 (autophagy-related 8) degradation assay [45,151].

### 3.6. Differentiation and Morphology

The Hedgehog pathway has well-established roles in the development and maintenance of high eukaryote differentiation. In yeasts, changes in morphology and differentiation occur at two very distinct levels. Individual cells can differentiate by changing shape, shifting from yeasts to very elongated polarised cells of true or pseudo hyphae. Colonies can display multiple 3D shapes [152] that relate with the ability to invade and adhere. At this level, *Candida* species, namely *C. albicans*, have been more studied than *S. cerevisiae*, mainly due to their pathogenicity. 

*C. albicans* is a commensal constituent of normal human microflora that acts as an opportunistic pathogen causing infections such as denture stomatitis, thrush and urinary tract infections, but can also provoke more severe systemic infections, in particular in immunocompromised individuals [153,154]. Among the underlying virulence mechanisms, the most well studied so far is probably the morphological switch from budding yeast to filamentous fungus, which, together with cell surface expression of adhesins and invasins (reviewed by [153]), allows colonisation of host tissues and their aggressive invasion, eventually overcoming the endothelial barrier [155]. The infection-associated yeast fast reproduction promotes formation of biofilms, not only on human and other organism tissues, but also on inert surfaces from clinical devices [154,156], constituting a major threat for hospitalised patients, in particular if immunocompromised.

**Figure 5:**
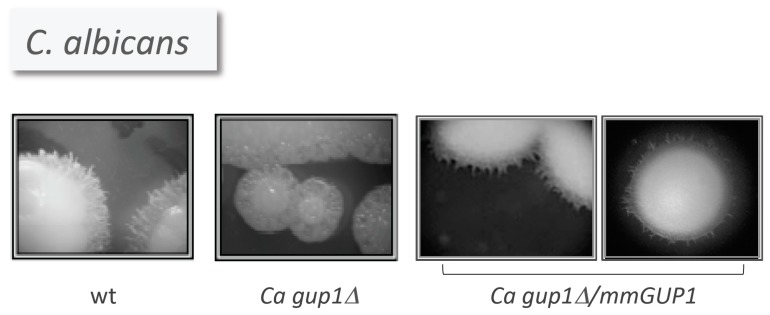
*Mus musculus* HHATL complementation of *C. albicans* colonies hyphae defect caused by the double deletion of *GUP1* alleles [29,156]. Pictures of colony borders were taken after 3 days of incubation in spider medium at 37 °C.

*C. albicans* has only one GUP orthologue: ca*GUP1*. caGup1 has been implicated in *C. albicans* virulence [29]. Accordingly, the *cagup1Δ* null mutant [157] was strongly affected in the ability to develop hyphae, to adhere, invade and form biofilm, and its colonies exhibited aberrant morphology/differentiation patterns [29]. Interestingly, *M. musculus* HHATL cDNA was able to complement the *cagup1Δ* null mutant morphogenic defects associated with colony invasive growth (Figure 5) [29]. Preliminary data suggests that the hyphae development defects of the mutant can be reverted although not completely by *S. cerevisiae GUP1*, as well as the human HHATL and *D. melanogaster* RASP(HHAT) [158]. This partial reversal corresponds to a notorious cell elongation into pseudohyphae instead of true hyphae. The amino acid sequences of *C. albicans* and *M. musculus* Gup proteins share only 20% similarity, whereas *S. cerevisiae* and *C. albicans GUP1* sequences share 58%. Moreover, as mentioned above, the two key MBOAT amino acid residues differ in yeast Gup1/2 and mammalian HHAT(L). It remains to be seen in the future how far the high eukaryotes HHAT(L) ability goes in complementing yeast phenotypes, namely using alternative expression strategies and if the substitutions in the MBOAT residues play a role.

Similarly to *S. cerevisiae*, deletion of *GUP1* in *C. albicans* modifies ergosterol distribution at the level of plasma membrane [29]. Among various lipids of yeast membranes, ergosterol has been used as target of the most common antifungals like polyenes and azoles [159,160]. In *S. cerevisiae*, deletion of *GUP1* causes high resistance to ergosterol synthesis inhibitors including azoles [37]. Likewise, *C. albicans cagup1Δ* null mutant displayed increased resistance to fluconazole, ketoconazole and clotrimazole [29].

Another important phenotype of the *cagup1Δ* null mutant is the delayed ability to form biofilm [29]. The formation of biofilms, as the formation of colonies, derives from extensive colonisation of the environment, generating multicellular structures that allow regular feeding of all cells, comparably to a proto-tissue. Traditionally, *C. albicans* or other closely related yeasts have been extensively used to study biofilm formation. *S. cerevisiae* can be used to mimic biofilm by promoting its growth onto large communities such as giant colonies or mats [2,161,162,163]. Analogously to the tissues from higher eukaryotes, these multicellular aggregates depend on the production and secretion of extracellular matrix (ECM) [163]. In *S. cerevisiae*, this was shown to be made of two different polysaccharides [10] and to harbour a large and abundant proteome composed of 684 well-identified proteins belonging to very different functional classes [11]. The deletion of *GUP1* in *S. cerevisiae* provokes a profound difference in the composition of the secretome, yielding a sludgy-textured ECM that lost one of the polysaccharides present in wild-type ECM and harbours approximately 15% less proteins, 26% of which are not found in the wild-type ECM [10,11]. Additionally, DIGE analysis identified as well common proteins though present in statistically different amounts [11]. Among the missing proteins, there are some key regulators of metabolic pathways, namely Fbp1, Pyc2 and Pdc5/6 from glycolysis/gluconeogenesis/fermentation; important effectors in cytoskeleton organization like Tub2, Ent2/3, Rvs161 or VPS; stress response and secretory pathway proteins like Vma6/7, Vph1 or Ctt1 and Ecm4/38; and proteins involved in ubiquitination and sumoylation [11]. Moreover, the selective presence in the wild-type ECM of alpha-saccharides remodelling enzymes, such as Glc3 1,4-α-glucan branching enzyme, Mnn2 α-1,2-mannosyltransferase and Sga1 α-glucoamylase, suggest the presence of α-based polysaccharides in opposition to the cell wall beta sugars backbone. These seem to be important for ECM texture as inferred from the absence of these enzymes in *gup1∆* mutant ECM [11]. On the other hand, the mutant ECM comprises a great number of proteins involved in cell wall remodelling that were not found in the wild-type ECM, including the homologous GPI-anchored putative mannosidases Dcw1 and Dfg1, required for cell wall biosynthesis, Utr2, Kre6 and Krt2 proteins involved in the biosynthesis of β-glucans, Pir1 and Pir2 proteins involved in stabilization of the cell wall, and GPI-anchored protease Yps1 required for cell wall growth and maintenance. Clearly *GUP1* considerably affects the secretion of both glycosides and proteins into the yeast ECM.

## 4. Proteins Interacting with Yeast Gup1 and Gup2

Although much work is still necessary to fully identify the full interactome of Gup1/2 proteins in yeast, these have putative physical partners, predicted by whole genome screenings, or ascertained in particular single-gene studies in the case of Gup1 (Table 3). Gup2 partners include interesting proteins (Table 3), like Ste2, the receptor for α-factor, a yeast pheromone that initiates signalling response to mating by interacting both with the pheromone and a heterotrimeric G-protein [164]. On the other hand, Gup1 co-localises with Mep2 [165] and Por1 [45] and immunoprecipitates with Por1 and Pil1 [45]. Pil1 is a major constituent of eisosomes, and Por1 is the mitochondrial VDAC (voltage-dependent anion channel). The nature of these two last interactions remains unknown for the time being. Biochemically, Por1 interacts with Sfk1, a suppressor of the calcineurin inhibitor immunosuppressant drug FK506, by which it modulates the mitochondrial ionic balance [166]. *gup1∆* is resistant to FK506 [65], suggesting that Gup1 might be necessary for the interaction of Por1 with Sfk1, which would therefore be impaired from inhibiting FK506 in the mutant.

Mep2 belongs to a highly conserved Mep/Amt/Rh superfamily that also includes the Rh blood-group human antigens [167]. There are three well-characterised Mep-type ammonium transporters in *S. cerevisiae*, Mep1, 2 and 3 [168]. *S. cerevisiae* Mep2 and its positive regulator Npr1 kinase [169,170], and *C. albicans* Mep1 and Mep2 [171], are indispensable for differentiation, i.e., for true or pseudo-hyphae formation respectively. Mep2 has accordingly been associated with TORC1 signalling, implicating in CLS and ageing [172]. Moreover, Mep2 promotes activation of the PKA pathway by ammonium on nitrogen-starved cells [173]. *GUP1* is required for proper distribution of Mep2 over the plasma membrane, while another protein that also putatively interacts with Gup1, Vma4 (Table 3), is required for Mep2 expression [165]. In *gup1∆*, Mep2 is localised in patches instead of homogenously over the entire plasma membrane, while both transport and signalling associated to Mep2 function are increased [165]. Although these authors suggest that the effect of Gup1 on Mep2 might be indirect in view of the multiple phenotypes associated with *GUP1* gene involving plasma membrane, rafts and wall, it seems more likely that Gup1 directly negatively regulates Mep2. This would justify the higher Mep2-mediated transport and signalling observed in the strain deleted in *GUP1* (with Mep2 uninhibited). However, Mep2 is released naturally onto the wild-type yeast ECM [11], suggesting that the picture is much more complex. 

## 5. Gup1/2 Homologues from High Eukaryotes

### 5.1. Hedgehog Pathway

Yeast Gup1 and its close homologue Gup2 share significant sequence homology with proteins belonging to higher eukaryotes, from *Caenorhabditis elegans*, to *Drosophila melanogaster*, to mammals. All of those proteins, identical to Gup1/2 from yeast [23], were identified as members of the membrane bound *O*-acyltransferase (MBOAT) family based on their sequence homology. They all share two highly conserved regions of amino acids: one hydrophobic region containing an invariant histidine and a region composed of moderately hydrophobic amino acids that surround a well-conserved asparagine/aspartic acid residue [23]. The only exception found so far is the mammalian homologue of Gup1 HHATL (Hedgehog acyltransferase-like), in which the homologous regions are maintained, but the conserved histidine residue is replaced with a leucine. Thanks to the high degree of conservation of these two regions, at least 11 members of the MBOAT family have been identified so far in the human genome [38].

MBOAT family proteins also share a common topology. They all have 8–12 TMDs and localise in the ER or Golgi [38,181,182]. HHAT was first predicted to have eight TMDs [40], but this topology seems inconsistent when compared to other members of the MBOAT family. Specifically, the MBOAT signature amino acid residues would be located in the cytoplasm, which would be a feature unique to HHAT among all the other MBOAT members [42]. Recently, the topology of human HHAT has been further investigated using different prediction software, and the results obtained by two independent research groups showed that HHAT contains 10 TMDs and two re-entrant loops. The invariant His is located in the lumenal side of the endoplasmic reticulum, whereas the Asp residue is on the opposite side [42,183]. Conceivably, HHATL should have a similar structure, due to the high sequence similarity.

Most members of the MBOAT family catalyse the transfer of long chain fatty acids to hydroxyl groups of other lipophilic molecules [184,185,186], or the formation of wax esters [187]. They are categorised into three subgroups based on their biochemical reaction, due to the variety of processes in which they are involved [38]. Mammalian HHAT and its fly counterpart, together with Porcupine and Ghrelin *O*-acyltransferase (GOAT), belong to the group involved in the acylation of secreted proteins [38,182]. In particular, HHAT is the protein responsible for the N-palmitoylation of the Hedgehog morphogens [41,42] and Spitz (also known as Torpedo or DER), a fly protein homologous to TGF-alpha, that either acts as a secreted or as a cell-bound EGFR ligand [42]. *M. musculus* and human HHATL, as mentioned above, have the invariant His necessary for the palmitoylation activity of HHAT replaced by a Leu [40,42]. It is, therefore, expected to not act as an acyltransferase, although it was found to co-localise with both HHAT and Sonic in the ER [21]. It was shown that HHATL interacts directly with Shh decreasing the efficiency of Shh palmitoylation [22], being proposed as a negative regulator of the HHAT-driven N-palmitoylation of Shh and of the Hh pathway. However, the precise mechanism of action has yet to be described.

In mammals as well as in *Drosophila*, HHAT promotes Hedgehog secreted morphogens N-palmitoylation [20,21,22,40]. Hh morphogens are diffusible proteins involved in long distance cell-cell communication, playing fundamental roles in embryonic development, patterning and cellular differentiation [188,189,190,191], as well as in adult tissue homeostasis and cancer [192]. Proper signalling is achieved by a specific gradient of Hh morphogens concentration from the cells that produce it to those destined to bind them [169,193,194,195]. Hh post-translational lipophilic modifications have a key role in maintaining the protein concentration gradient, influencing the long range signalling [196,197], which depends on the formation of diffusible multimeric forms [198,199,200]. On the other hand, secretion of Hh morphogens might operate through several mechanisms. One is the formation of specific fat body-derived diffusible lipoprotein particles [196,201], which in *Drosophila* largely depends on lipid rafts [202,203,204]. Another mechanism is an exosome-mediated pathway [205,206]. Exosomes derive from the endocytic compartment where vesicles are packed [207]. Yeasts also produce and secrete exosomes [208,209], which proteome was characterised in *C. albicans* [210,211]. Still there is no idea regarding their biological roles.

In mammalian cells, Hedgehog responsive cells activate transcription through Gli1. This occurs in the Hh responding cells by the triangulation Hh ligand-Ptch1-Smo. Gli1 can be activated by Smo-independent mechanisms, increasing Hh signalling even in the absence of the Hh ligand. This non-canonical activation of Gli1 was described in association with oesophageal adenocarcinoma [212]. In that case, Gli1 is controlled by a TNFα-derived TORC1/S6K1 pathway. TORC1 promotes the phosphorylation of Gli1 (at S^384^) by S6K1, freeing it from SuFu and easing the entrance to the nucleus. Additionally, TORC2 promotes the stabilisation of Gli1 by keeping it phosphorylated (at S^230^) through the action of AKT [212,213]. AKT commands a *star*-type pathway and demands intracellular signalling by phosphoinosides, from which the designation of PI3K-PKB/AKT pathway (reviewed by [214]). Other possible cross-talks with Hedgehog have been suggested to operate at the level of Gli1 activation: MAPK/ERK [215] and KRAS [216]. 

### 5.2. HHAT(L) Proteins Expression and Associated Pathologies

Palmitoylation at the N-terminal domain of Hedgehog proteins is necessary for the proper Hh signalling at both long and short range [217]. It was shown in vitro that palmitoylated forms of Shh are 40–160-fold more active compared to unmodified Shh [218]. Palmitoylated Sonic Hh is required for the formation of multimeric complexes that are fundamental for extracellular migration [199]. Therefore, the proper functionality of HHAT is fundamental for maintenance of the correct Hh function. This should stand for the Hh pathway function, namely during embryogenesis. Accordingly, HHAT loss of function by a single G287V missense mutation resulted in serious sex development defects [219]. In general, Hh signalling malfunction, causes severe and often life-threatening developmental disorders such as holoprosencephaly, craniofacial defects and skeletal malformations [31,220,221,222]. 

In human tissues, the HHAT(L) picture appears somewhat more complex than predicted [22]. The HHATL gene is expressed in very few tissues and in different amounts: heart > skeletal muscle > brain [34,35,223]. Actually, the gene maps to a locus of dilated cardiomyopathy (CMD1E/OMIM 601154), a disorder characterised by ventricular dilation and impaired systolic function, resulting in congestive heart failure and arrhythmia, eventually causing premature death that also harbours another gene encoding a cardiac muscle Na^+^ channel (SCN5A/HH1). Despite this association, the absence of HHATL disease-specific mutations in a cohort of 48 patients [34], leaves no clear indication that HHATL might be responsible for the disease.

It has been estimated that 25% of all human tumours required Hh signalling to maintain tumour cell viability [167]. Corrupted Hh signalling, namely through over-activation, underlies many types of cancer, including small and non-small cell lung cancer, squamous cell and basal cell carcinomas and myeloid leukaemias [224,225,226,227,228]. The importance of Hh signal palmitoylation on this regard has recently been demonstrated. The knockdown of HHAT caused in vitro reduced proliferation and invasiveness of human pancreatic ductal adenocarcinoma [229]. Therefore, it is conceivable that blocking the Hedgehog pathway using HHAT inhibitors may block tumour progression [230,231]. Moreover, HHATL transfected cells presented lower capability of cell proliferation, invasion and tumorigenicity, supporting the notion that the protein might function as a tumour suppressor [36]. This function is compatible with the negative regulation of HHAT-mediated Hh palmitoylation described for HHATL [22]. The reason why this gene is only expressed in few tissues appears intriguing. Other works reported the expression of KIAA1173 (another *GUP1*/HHATL alias—Table 1) in normal skin [232] and nasopharyngeal mucosa [36], and its extreme down-regulation in cells of skin squamous cell carcinoma [232]. Still, the KIAA1173 protein is N-172 amino acids shorter than the cardiac isoform above-mentioned [34], suggesting that the HHATL ORF might undergo extensive RNA or protein splicing and perform different roles in different tissues.

The HHAT gene has also been associated with type 2 diabetes [233]. Its changed expression was found in patients undergoing bariatric surgery [233], further suggesting a correlation with glucose homeostasis and therefore obesity. *Diabetes mellitus* and obesity are closely related [234], through deficient glycerol consumption by glycerol kinase [235] and deficient tissue regulation of glycerol transport by aquaglyceroporins [236]. This once again raises the notion that in yeasts Gup1 implicates in glycerol transport, consumption and production for osmoregulation purposes [19,28] and that it apparently connects nutrient regulation to morphogenic-related Hedgehog-like phenotypes, through a signalling entanglement between the CWI/Pkc1, TORC2/YPK TORC1/Pkh1/2 and HOG/Sho1 pathways.

## 6. Similarity Analysis of the GUP/HHAT(L) Amino Acid Sequences

Gup proteins from both yeast and higher eukaryotes belong to the MBOAT superfamily of membrane-bound *O*-acyltransferases, although only the mammalian HHA*T* has been assigned a function as an enzyme performing the palmitoylation of the Sonic Hedgehog ligand [21]. All eukaryotes have at least one such gene/protein. Therefore, the databases present an extremely large number of sequences designated as Gup or HHAT(L). This generalised presence does not necessarily mean that the amino acid sequence is highly conserved. Multiple sequence alignment similarity matrices were built using Clustal Omega [237], based on the accession numbers available at UniProt database and confronting them with NCBI available sequences (Supplementary File S1). These were grouped in a simple manner: yeasts, filamentous fungi, mammals, fish, teleostomi, reptiles, birds, amphibian, insects, nematodes, testudines and arthopoda (Supplementary File S2). A few isolated organisms presenting GUP/HHAT(L) sequences were grouped as *others* in Supplementary File S2. Gup1/HHATL is absent from nematodes and insects, while Gup2/HHAT is absent from reptiles, amphibian, testudines, teleostomi and *Zea mays*, the only plant where a Gup amino acid sequence was so far identified. Within each matrix, the percentage of similarity varied between two extreme values (shadowed in File S2). Notably, Gup1/HHATL sequences from nematodes are the most diverse. Generally, Gup1/HHATL sequences are less diverse than the Gup2/HHAT, which is perceptible in the respective cladograms (Figure S1A,B) built using Mega 7 (Molecular Evolutionary Genetics Analysis) [238]. Still, the difference in similarity between the Gup1/HHATL and Gup2/HHAT sequences is very small. As an example, we present in Table 4 the similarity matrix between the better studied Gup proteins: *S. cerevisiae* Gup1 and Gup2, *C. albicans* Gup1, *M. musculus* HHATL and HHAT, *H. sapiens* HHATL and HHAT and *D. melanogaster* RASP/HHAT. The sequence similarity presented is very high. *M. musculus* HHAT shares 20% similarity with yeast Gup2 and 19.3% with yeast Gup1, while HHATL shares 21.9% similarity with yeast Gup2 and 20.7% with yeast Gup1. The frontier between proteins considered HHAT and HHATL is very narrow. Moreover, some sequences actually appear to be too different from the yeast Gup proteins to even be considered as such (Supplementary File S2).

## 7. Conclusions

Paracrine signalling has not yet been recognised as occurring in microbial communities. However, the notion that microbes multicellular aggregates can be regarded as tissues, suggested for the first time by Shapiro in 1988 (reviewed by [239]), predicts that each individual cell does not live exclusively in response to environmental stimuli, as happens in planktonic life, but must involve cell–cell communication. It remains unclear whether this may happen through a diffusible chemical, like ammonia [12,240,241] or the quorum-sensing chemicals [13,14], or through a peptide signal like in higher Eukaryotes. The large number and diverse proteome from the yeast ECM [11,210,211] hampers the identification of a putative peptide signal. Additionally, the also high number of proteins identified as being differently expressed in the *GUP1*-deleted mutant ECM compared to the wt strain, does not allow the suggestion of a candidate [11]. Nevertheless, the fact that all Eukaryotes present a Gup/HHAT(L) protein suggests a conserved mechanism in which these proteins may participate. One should, however, not discard the hypothesis that proteins from the same group might perform very different roles in different organisms. The plethora of phenotypes from the yeast *gup1∆* mutant, and the multiple localisation of the Gup1 protein, actually favour this as having multiple roles.

**Figure 6:**
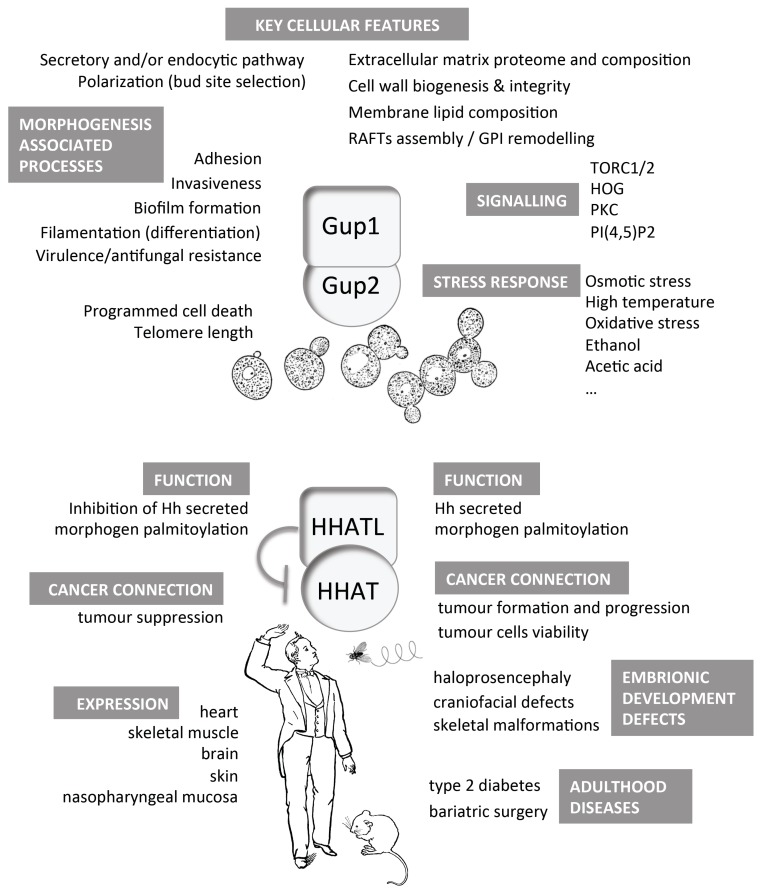
Phenotypes and biological processes associated to the Gup proteins in yeasts (*S. cerevisiae* and *C. albicans*) and in the higher eukaryotes *M. musculus*, *H. sapiens* and *D. melanogaster*. Clipart was downloaded from the free access supplier http://etc.usf.edu/clipart/home/.

The most prominent Gup1 and Gup2 localisation is the plasma membrane [19,41,45]. In mammalian or fly cells, HHAT is almost exclusively located in the ER, while the localisation HHATL is still scarcely documented. Data suggest that yeast Gup1 is, or locates at, a hub between CWI/Pkc1, TORC2/YPK, TORC1/Pkh1/2 and HOG/Sho1 pathways, controlling response to nutrients and stress and morphogenic-related Hedgehog-like phenotypes. Therefore, it is not surprising that *GUP1* deletion-associated phenotypes cover a vast number of pathways controlling basic processes of yeast life (summarised in Figure 6), namely associated with growth and development and that the deletion of the gene does not, in spite of its central role, become lethal. Altogether only fitness appears to be decreased in most of the environmental conditions studied (temperature, pH, stress and so on).

Importantly, most of the phenotypes caused in yeast by the deletion of *GUP1* or *GUP2* are associated with the *GUP1* deletion, in opposition of mammalian and fly cells in which case the information available almost exclusively regards Gup2/HHAT (Figure 6). This does not mean that Gup2 has no function in yeasts. In mammals, the Gup2/HHAT is the enzyme that performs the palmitoylation of the Hh signal [21], while Gup1/HHATL acts as an inhibitor of that palmitoylation [21] through an as yet unknown mechanism. If that would be the case for yeasts, Gup2 could be performing a role that is inhibited by Gup1 and, therefore, all of the phenotypes associated with the deletion of *GUP1* would actually derive from the uninhibited function of Gup2.

A last comment goes to nomenclature. It is important that Gup proteins are not further taken as glycerol transporters. This mistaken allusion has equally appeared in the literature dedicated to yeasts or mammalian cells. Due to the poor understanding of these proteins, the Gup1 from *S. cerevisiae* has even been cloned and heterologously expressed as if it were the actual glycerol permease (e.g., [27]). Moreover, careful naming would be recommended in the fields of research using high eukaryotes, in view of the extreme similarity between HHAT and HHATL and between these and the original *S. cerevisiae* Gup1 and Gup2 proteins. In order to avoid the misguidance generated by the multiple designations (Table 1) we propose a single notification. Based on the examples of the Ras and TOR proteins, for which the name used in the higher eukaryotes and the numeration of yeasts were combined, we propose that HHAT1 and HHAT2 are consistently adopted hereon to be designated Gup1/HHATL and Gup2/HHAT, regardless of the biological system under study.

A lot is still required to unveil the true nature and biological function of the Gup proteins. The results so far contribute to stress that studies based on either yeasts or higher eukaryotes are equally important and complement themselves. In particular, in the near future, the search for the molecular partners/targets of the Gup proteins in yeast will be important, along with an understanding of why Gup1/HHATL is only present and important in a few adult human tissues, the physiology of which does not straightforwardly relate to the Hedgehog pathway.

## Figures and Tables

**Table 1:** Gup1 and Gup2 aliases in the literature and databases.

Aliases		Organism	Key References
Gup1	Gup2	*Saccharomyces cerevisiae*	[19,28]
Gup1	-	*Candida albicans*	[29]
-	RASP	*Drosophila melanogaster*	[30]
	Skinny Hedgehog		[20]
	Sightless		[31]
	Central Missing		[32]
HHATL	HHAT	*Mus musculus*	[22]
HHATL	HHAT	*Homo sapiens*	[33]
c3orf3			[34]
KIAA117			[35,36]
MBOAT3 *			[33]
MSTP002 ^#^			[33]
OACT3 *			[33]

* Nomenclature shared with *Xenopus laevis* and *X. tropicalis*; ^#^ Nomenclature shared with *X. tropicalis*.

**Table 2:** Documented transcription factors that function as regulators of Gup1 and Gup2 [46]. Activ.: activator; Inhib.: inhibitor.

Predicted Function in *GUP1* and *GUP2* Transcription Regulation											
*GUP1*				*GUP2*			Identical Regulation			Opposite Regulation	
Activ.	Inhib.	Both	Activ.		Inhib.	Both	Activ.	Inhib.	Both	Activ. *GUP1* + Inhib. *GUP2*	Inhib. *GUP1* + Activ. *GUP2*
Ash1	Abf1	Rap1	Ecm22	Sfp1	Ace2	Aft1	Tec1	Mig3	Rap1	Ash1	none
Met4	Cac2	Spt23	Gat4	Snf2	Ash1	Cbf1					
Tec1	Cup9	Yrm1	Gcr2	Snf6	Bas1	Cin5					
	Gal4		Gis1	Sok2	Fhl1	Plm2					
	Hir3		Gln3	Spt10	Gcn4	Pho4					
	Mig3		Gzf3	Spt2	Gcr2	Rap1					
	Sfp1		Hap5	Ste12	Mig3	Rfx1					
	Sum1		Hda1	Sut1	Msn4	Sko1					
	Uls1		Hms1	Swi3	Rif2	Xbp1					
			Isw2	Taf14	Set2	Yap6					
			Mbp1	Tec1	Swi5	Msn2					
			Mcm1	Tup1	Tbf1	Swi4					
			Mga1	Uls1							
			Mig1	Uc2							
			Mot3	Urc2							
			Nrg1	Xbp1							
			Rgm1	Yhp1							
			Rox1	Yox1							
			YR015C								

Observations: Ash1: TF inhibitor of HO transcription; Mig3: TR with a major role in catabolite repression and ethanol response; inactivated by phosphorylation by Snf1 or Mec1 pathway; Rap1: Essential TR for many loci: transcription activation and repression, chromatin silencing, and telomere length maintenance; Tec1: TF targeting filamentation genes and Ty1 expression; activity dependent on association with Ste12; Ste12 activation of most filamentation gene promoters depends on Tec1.

**Table 3:** Gup1 and Gup2 ascertained and predicted physical partners.

	Partner	Assay	Type of Study	Key Reference	Assigned Function
Gup1	Fet3	PCA	HTP	[174]	Iron-O_2_-oxidoreductase; multicopper oxidase that oxidizes ferrous (Fe^2+^) to ferric iron (Fe^3+^) for subsequent cellular uptake by transmembrane permease Ftr1.
	Frk1	BA	HTP	[175]	Protein kinase of unknown cellular role; interacts with rRNA transcription and ribosome biogenesis factors, and the long chain fatty acyl-CoA synthetase Faa3p.
	Hek2	AC	HTP	[176]	RNA-binding protein involved in asymmetric localization of ASH1 mRNA; represses translation of ASH1 mRNA, an effect reversed by Yck1-dependent phosphorylation; regulates telomere position effect and length.
	Mep2	2H/Co-L	Single study	[165]	NH4+ permease; regulation of pseudohyphal growth; expression regulated by nitrogen catabolite repression (NCR).
	Msc7	PCA	HTP	[177]	Cytoplasmic protein of unknown function.
	Nab2	AC	HTP	[178]	Nuclear poly(A)-binding protein; required for nuclear mRNA export and tail length control.
	Pil1	Co-IP	Single study	[45]	Eisosome core component; detected in phosphorylated state in mitochondria; phosphorylated upon Pkc1 hyperactivation in a Slt2p MAPK-dependent fashion.
	Por1	Co-IP/Co-L	Single study	[45]	Mitochondrial voltage-dependent anion channel (VDAC); required for maintenance of mitochondrial osmotic stability and membrane permeability; couples the glutathione pools of the intermembrane space and the cytosol.
	Sat4	BA	HTP	[175]	Ser/Thr protein kinase involved in salt tolerance; functions in regulation of Trk1-Trk2 potassium transporter.
	Vtc4	PCA	HTP	[174]	Vacuolar membrane polyP polymerase; subunit of the vacuolar transporter chaperone (VTC) complex; regulates membrane trafficking; role in non-autophagic vacuolar fusion.
	YHL042W	PCA	HTP	[174]	Putative protein of unknown function.
Gup2	Aqy1	PCA	HTP	[174]	Spore-specific aquaporin.
	Aus1	PCA	HTP	[179]	Plasma membrane sterol transporter of the ATP-binding cassette family; required, along with Pdr11, for uptake of exogenous sterols and their incorporation into the plasma membrane.
	Hsp30	PCA	HTP	[174]	Negative regulator of the H(+)-ATPase Pma1; stress-responsive; induced by heat shock, ethanol, weak organic acid, glucose limitation, and entry into stationary phase.
	Ifa38	PCA	HTP	[174]	Microsomal beta-keto-reductase; mutants exhibit reduced VLCFA synthesis, accumulate high levels of dihydrosphingosine, phytosphingosine and medium-chain ceramides.
	Nam7	AC	HTP	[180]	ATP-dependent RNA helicase.
	Pdr10	PCA	HTP	[179]	ATP-binding cassette (ABC) transporter; multidrug transporter involved in the pleiotropic drug resistance network; regulated by Pdr1p and Pdr3p.
	Pho88	PCA	HTP	[174]	Probable membrane protein involved in phosphate transport; role in the maturation of secretory proteins.
	Sss1	PCA	HTP	[174]	Subunit of the Sec61 translocation complex (Sec61-Sss1-Sbh1); this complex forms a channel for passage of secretory proteins through the ER membrane.
	Ste2	PCA	HTP	[174]	Receptor for α-factor pheromone; interacts with both pheromone and a heterotrimeric G-protein to initiate the signaling response that leads to mating.

HTP: high throughput; PCA: protein complementation assay; AC: affinity capture (RNA); BA: biochemical activity; 2H: two-hybrid; Co-IP: co-immunoprecipitation; Co-L: co-localization.

**Table 4:** Similarity matrix between the most studied GUP proteins.

	*C. albicans* SC5314 Gup1	*S. cerevisiae* S288c Gup1	*S. cerevisiae* S288c Gup2	*D. melanogaster* Gup2/RASP	*M. musculus* Gup1/HHATL	*H. sapiens* Gup1/HHATL	*M. musculus* Gup2/HHAT	*H. sapiens* Gup2/HHAT
*C. albicans* SC5314 Gup1	100.00	57.64	47.02	16.63	19.15	18.45	18.49	18.77
*S. cerevisiae* S288c Gup1	57.64	100.00	56.61	17.84	20.70	20.45	19.30	19.80
*S. cerevisiae* S288c Gup2	47.02	56.61	100.00	17.80	20.92	20.20	20.00	21.07
*D. melanogaster* Gup2/RASP	16.63	17.84	17.80	100.00	21.62	21.14	25.94	26.58
*M. musculus* Gup1/HHATL	19.15	20.70	20.92	21.62	100.00	90.07	28.16	27.92
*H. sapiens* Gup1/HHATL	18.45	20.45	20.20	21.14	90.07	100.00	28.33	27.86
*M. musculus* Gup2/HHAT	18.49	19.30	20.00	25.94	28.16	28.33	100.00	82.91
*H. sapiens* Gup2/HHAT	18.77	19.80	21.07	26.58	27.92	27.86	82.91	100.00

## Supplementary Materials

The following are available online at www.mdpi.com/2221-3759/4/4/33/s1.

## Nomenclature

According to the rules from Yeast scientific nomenclature, genes, proteins and mutants are presented in, capitals/italic (ex.: *GUP1*), title case (ex.: Gup1), and lower case/italics (ex.: *gup1∆*), respectively. In the case of other model organisms, proteins and genes are presented as in the corresponding literature.
