# Supplementary material for: Yeast Gup1(2) Proteins Are Homologues of the Hedgehog Morphogens Acyltransferases HHAT(L): Facts and Implications
Source: J Dev Biol. 2016 Nov 5;4(4):33. doi: 10.3390/jdb4040033 (PMC5831804; doi:10.3390/jdb4040033)
Supplement: Supplementary file 1 [file jdb-04-00033-s001.zip › Figure S1 Legend.pdf]

Supplementary Materials: Yeast Gup1(2) Proteins Are Homologues of the Hedgehog Morphogens Acyltransferases HHAT(L): Facts and Implications

Cândida Lucas, Célia Ferreira, Giulia Cazzanelli, Ricardo Franco-Duarte and Joana Tulha

Legend

Figure S1. Cladogram of the Gup1/HHATL (**A**) and Gup2/HHAT (**B**) using the sequences in Files S1 and S2.
