# Supplementary figures and images for: Yeast Gup1(2) Proteins Are Homologues of the Hedgehog Morphogens Acyltransferases HHAT(L): Facts and Implications
Source: J Dev Biol. 2016 Nov 5;4(4):33. doi: 10.3390/jdb4040033 (PMC5831804; doi:10.3390/jdb4040033)

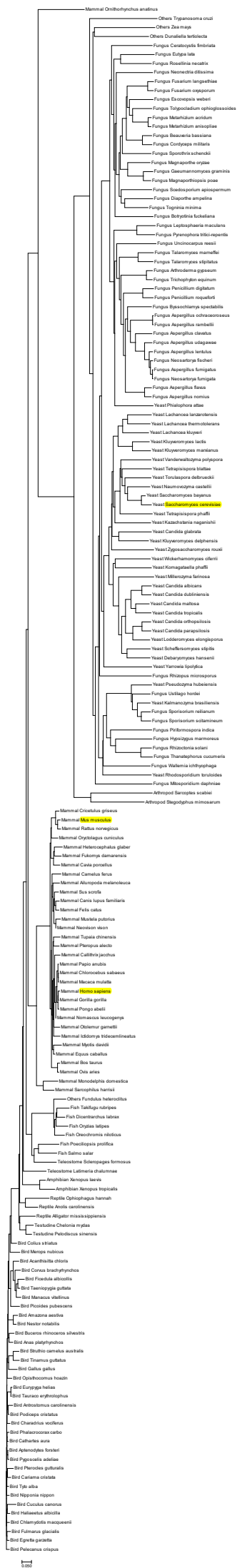

Supplement: Supplementary file 1 [file jdb-04-00033-s001.zip › Figure S1A.pdf]

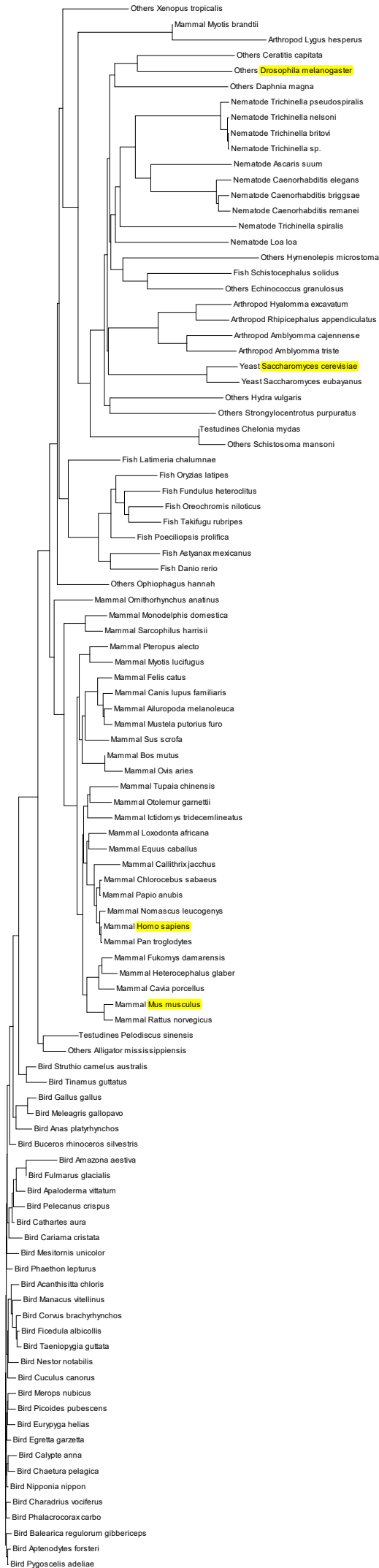

0.050

Supplement: Supplementary file 1 [file jdb-04-00033-s001.zip › Figure S1B.PDF]
